# Identification of a Novel OX40L^+^ Dendritic Cell Subset That Selectively Expands Regulatory T cells

**DOI:** 10.1038/s41598-018-33307-z

**Published:** 2018-10-08

**Authors:** Alejandra Marinelarena, Palash Bhattacharya, Prabhakaran Kumar, Ajay V. Maker, Bellur S. Prabhakar

**Affiliations:** 1 Department of Microbiology and Immunology, University of Illinois College of Medicine, Chicago, Illinois USA; 2 Department of Surgery, Division of Surgical Oncology, University of Illinois College of Medicine, Chicago, Illinois USA

## Abstract

We have previously shown GM-CSF derived bone-marrow dendritic cells (G-BMDCs) can induce the selective expansion of Tregs through the surface-bound molecule OX40L; however, the physiological role of this *ex vivo* derived DC subset remained to be elucidated. We determined GM-CSF administration to mice induced the generation of *in vivo* derived OX40L^+^ DCs, phenotypically similar to *ex vivo* OX40L^+^G-BMDCs, in the spleen, brachial lymph nodes and liver. The generation of OX40L^+^ DCs correlated with increased percentages of functionally suppressive Tregs in the spleen, brachial lymph nodes, and liver of GM-CSF treated mice. DCs from GM-CSF treated mice expanded Tregs in CD4^+^ T-cell co-cultures in an OX40L dependent manner, suggesting OX40L^+^ DCs may play a role in peripheral Treg homeostasis. Furthermore, comparing the transcriptome data of OX40L^+^ DCs to that of all immune cell types revealed OX40L^+^ DCs to be distinct from steady-state immune cells and, microarray analysis of OX40L^+^G-BMDCs and OX40L^−^G-BMDCs revealed higher expression of molecules that are associated with tolerogenic phenotype and could play important roles in the function of OX40L^+^ DCs. These findings suggest that OX40L^+^ DCs may represent a unique DC subset induced under inflammatory conditions that may play an essential role in maintaining Treg homeostasis.

## Introduction

Dendritic cells (DCs) comprise a heterogeneous population of antigen presenting cells which facilitate and regulate innate and adaptive immune response by initiating T-cell priming and differentiation. DCs are responsible for the capture, processing, and presentation of MHC bound antigenic peptides to T lymphocytes bearing cognate T cell receptors^1–4^. While DCs have been shown to play a vital role in the initiation of immune responses to pathogens, studies have also suggested a critical role for DCs in the maintenance of immune tolerance^5^. The specific deletion of CD11c, an integrin expressed at high levels by DCs, and at lower levels by the other cells of the innate immune system, has been shown to result in the induction of spontaneous autoimmunity, characterized by the infiltration of CD4^+^ T-cells into peripheral tissues, autoantibody formation, and onset of inflammatory bowel disease suggesting a role for CD11c^+^ DCs in the maintenance of immune tolerance^6^.

Since the depletion of DCs can lead to autoimmune pathologies, it has been postulated that increasing DC populations could restore tolerance and prevent autoimmunity. Injection of Fms like tyrosine kinase 3 ligand (FLT3L), a hematopoietic cytokine required for DC development, increased the proportion of DCs and subsequently prevented diabetes onset in NOD mice^7^. Similarly, we have previously reported the prevention and/or suppression of several experimental autoimmune diseases, such as type 1 diabetes^8^, autoimmune thyroiditis^9–11^, and myasthenia gravis^12–14^, upon treatment with granulocyte macrophage colony-stimulating factor (GM-CSF), another hematopoietic cytokine strongly linked to myeloproliferation as well as DC development. In each case, a significant increase in splenic Tregs was observed in GM-CSF treated mice. Interestingly, the increase in Tregs in GM-CSF treated mice corresponded with an increase in CD11c^+^CD8α^−^ DCs^11^. Further, we demonstrated that the therapeutic effect of GM-CSF was primarily mediated through the mobilization of CD11c^+^CD8α^−^ DCs that could stimulate the expansion of Tregs *in vivo* and suppress autoimmune disease through increased IL-10 production^10,11^. Interestingly, subsequent studies discovered that *ex vivo* derived DCs, generated from bone marrow (BM) precursor cells isolated from WT or MHC Class-II^−/−^ mice differentiated in the presence of GM-CSF (G-BMDCs), could selectively expand Foxp3^+^ Tregs in a cell-to-cell-contact dependent manner, independent of TCR-signaling, but most importantly, dependent on the DC cell surface expression of OX40L^15,16^.

OX40L, a member of the tumor necrosis factor superfamily, has been strongly implicated in the proliferation and survival of T cells by playing a critical role as a co-stimulatory molecule in association with T-cell receptor engagement^17,18^. Expression of this molecule has been detected on antigen presenting cells, such as dendritic cells^19^, B-cells^20^, and macrophages^21^, but can also be induced on various other immune cell types such as mast cells^22,23^, natural killer cells^24^, and vascular endothelial cells^25^. OX40L^+^CD11b^+^CD11c^+^ DCs have been identified in various autoimmune contexts such as in the pancreatic lymph nodes of NOD mice around the time of diabetes onset^26^, and in the inflamed kidneys of Lupus patients^27^, which suggested a role for OX40L in the pathogenesis of autoimmune diseases. OX40L has also been found to have genetic associations with multiple autoimmune diseases including Systemic Lupus Erythematosus (SLE)^28^, Systemic Sclerosis^29^, and, Sjogren’s syndrome^30^. Contrary to the previous studies, OX40L/OX40 interactions have also been reported in the homeostatic regulation of Tregs. A marked reduction in Treg numbers has been observed in the spleens of mice that are OX40 deficient while a marked increase in Treg numbers has been observed in the spleens of mice that overexpress OX40L^31–33^. Similarly, our findings have demonstrated a direct role for OX40L^+^ G-BMDCs in the selective expansion of Tregs, and not Teff cells, in the absence of canonical antigen presentation upon *ex vivo* co-culture with CD4^+^ T-cells^16^.

In this study we explore the function of OX40L^+^ DCs in physiological Treg homeostasis. Due to the scarcity of, and difficulty in, isolating DCs *in vivo* from tissues, most studies, including our laboratory, have long utilized bone-marrow progenitor culture systems with the use of hematopoietic cytokines such as GM-CSF^34^ or FLT3L^35^ to generate bone-marrow derived DCs (BMDCs). However, recent studies have suggested that DCs generated from bone-marrow precursor cells differentiated by GM-CSF may lack physiological counterparts *in vivo*^36^. While Flt3L deficiency severely affects the development of all types of DCs *in vivo*, mice lacking GM-CSF or its receptor do not show a severe defect in DC development^37–39^ suggesting *ex vivo* generated G-BMDCs may not have physiological counterparts in the steady-state^36,40^. Thus, it is proposed that GM-CSF may be required for the development and differentiation of a special class of DCs termed inflammatory DCs, described as DCs that may not be present in the steady-state, but readily available during conditions of inflammation or infection^41^.

To better understand the nature of tolerogenic OX40L^+^ G-BMDCs and to identify a relevant biological functional equivalent, we first investigated the function of *ex vivo* derived OX40L^+^ DCs by analyzing their cell surface marker and gene expression profiles. Furthermore, upon administration of GM-CSF to C57BL/6 mice, we observed elevated numbers in peripheral Tregs that correlated with increased OX40L^+^ DCs within the same tissues. Furthermore, isolation of OX40L+ DCs from GM-CSF treated mice and subsequent co-culture with CD4^+^ T-cells showed an increase in the Treg proliferation which we found was dependent on OX40L signaling. Finally, comparing the transcriptome data of OX40L^+^ DCs to that of other immune cells from the ImmGen database, suggested OX40L^+^ DCs to be of non-steady state identity. Collectively, our data suggest that OX40L^+^ DCs may represent a non-steady-state myeloid DC subtype involved in Treg expansion *in vivo* and point to a hitherto undiscovered mechanism of Treg homeostasis under inflammatory conditions where GM-CSF levels are expected to be much higher.

## Results

### OX40L^+^ G-BMDCs selectively expand phenotypically functional Tregs *ex vivo* with minimal expansion of the Teff compartment

**Figure 1:**
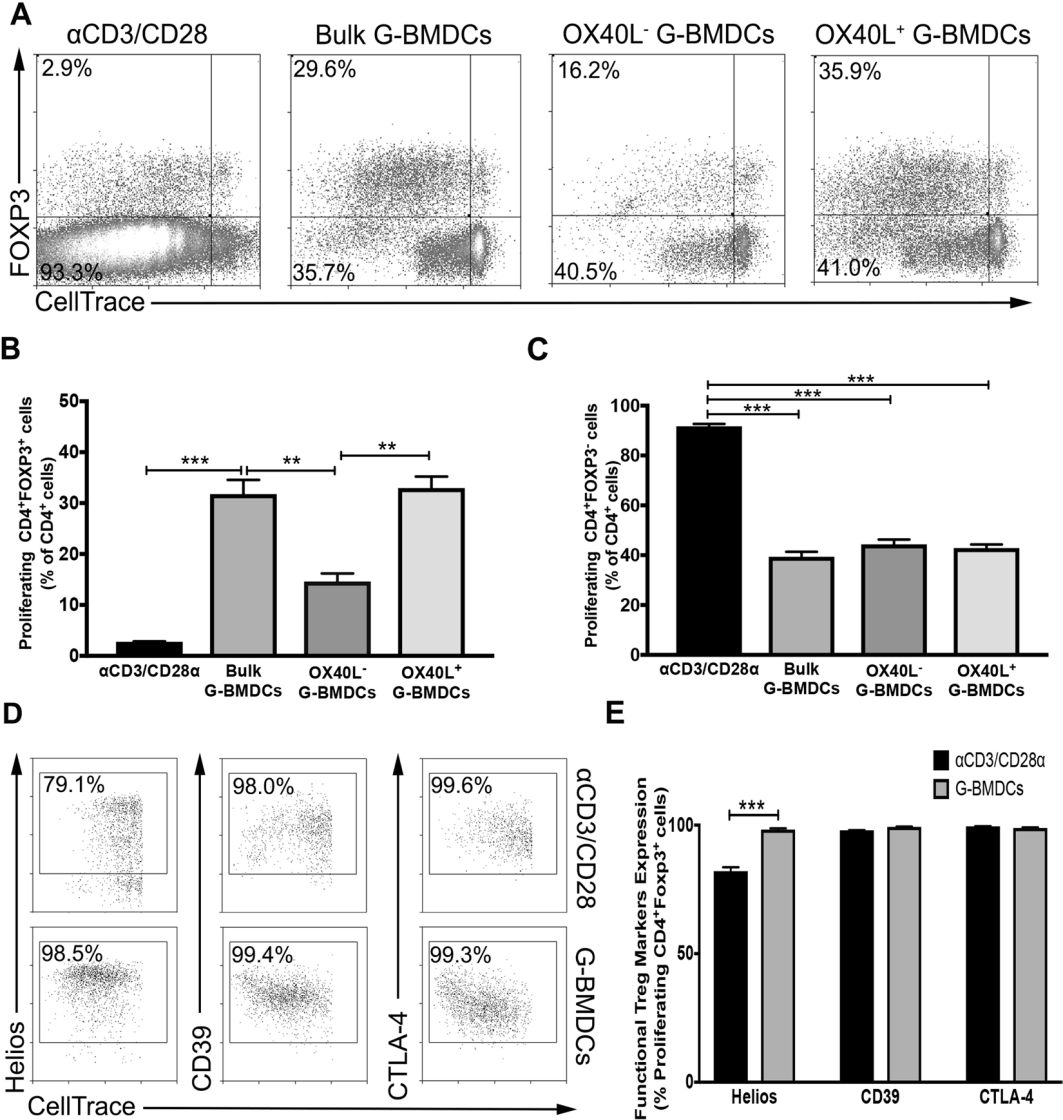
OX40L^+^CD11c^+^ G-BMDCs are responsible for the expansion of functional Tregs. Splenic C57BL/6 CD4^+^ T-cells were stained with CellTrace and separately co-cultured with anti-CD3/CD28 microbeads, bulk G-BMDCs (pre-sort) population, sorted OX40L^−^CD11c^+^ G-BMDCS, and sorted OX40L^+^CD11c^+^ G-BMDCS. (**A**) Representative dot plots of percent proliferating Tregs using CD4^+^FOXP3^+^ as a marker for Tregs. All flow cytometry plots were gated on CD4^+^ cells. (**B**,**C**) Bar graphs showing percent proliferation of Tregs and Teff in CD4^+^ T-cell co-cultures with indicated cell populations, (n = 3). The data show the means ± standard error of the mean (SEM). A p-value < 0.05 was considered significant; **p < 0.01, ***p < 0.001. (**D**) Representative dot plots of Treg suppressive markers on proliferating Tregs. (**E**) Bar graphs showing percent expression of Treg suppressive markers on proliferating Foxp3^+^ Tregs. Values are expressed as means ± SEM (n = 3; ***p < 0.001).

In order to elucidate the critical role of OX40L expression on CD11c^+^ cells in the expansion of Tregs, we sorted OX40L^+^ G-BMDC and OX40L^−^ G-BMDC populations from *ex vivo* expanded G-BMDC cultures and assessed their capacity to expand Foxp3^+^ Tregs upon co-culture with CellTrace labeled CD4^+^ T-cells. We compared this capacity to an alternative and common method of expanding Tregs *ex vivo* by employing TCR stimulation and co-stimulation using anti-CD3/CD28 T-activator beads. Cultures supplemented with anti-CD3/CD28 exhibited a modest Treg proliferative response (Fig. 1A,B) with a prominent increase in the percentage of proliferating Teff cells (CD4^+^FoxP3^−^) (Fig. 1A,C). In contrast, CD4^+^ T-cells cultured with unsorted G-BMDCs exhibited a significant increase in the percentage of Tregs (Fig. 1A,B) with minimal expansion of the Teff cells (Fig. 1A,C). Interestingly, while we observed a significant decrease in Treg proliferation in the presence of OX40L^−^ G-BMDCs relative to bulk G-BMDCs, we observed a further increase in Treg proliferation when CD4^+^ T-cells were co-cultured with OX40L^+^G-BMDCs, implicating the significant role of OX40L expressed on CD11c^+^ cells in the selective Treg expansion (Fig. 1A,B).

OX40 has been previously shown to expand Tregs *in vivo*, as a potential therapeutic for autoimmune disease, however, Tregs expanded by this mechanism have been shown to exhibit impaired suppressive function due to exhaustion^33^. Therefore, we examined the expression of various suppressive Treg surface markers on proliferating Tregs expanded by G-BMDCs. Compared to proliferating Tregs stimulated by anti-CD3/CD28, Helios, a transcription factor that stabilizes the Treg suppressive phenotype^42^, was expressed in a significantly higher percentage of proliferating Tregs in the G-BMDC co-cultures (Fig. 1D,E). Furthermore, we also assessed the expression of Treg suppressive markers CD39, an ectonucleotidase, and CTLA-4, a co-stimulatory molecule^43,44^. Both these molecules, highly implicated in Treg suppression function, were expressed in similar high percentages of proliferating Tregs in both the G-BMDC and anti-CD3/anti-CD28 stimulated cultures (Fig. 1D,E), suggesting Treg proliferation most likely did not impair the suppressive activity of expanded Tregs.

### OX40L^+^ G-BMDCs highly express co-stimulatory and co-inhibitory molecules

**Figure 2:**
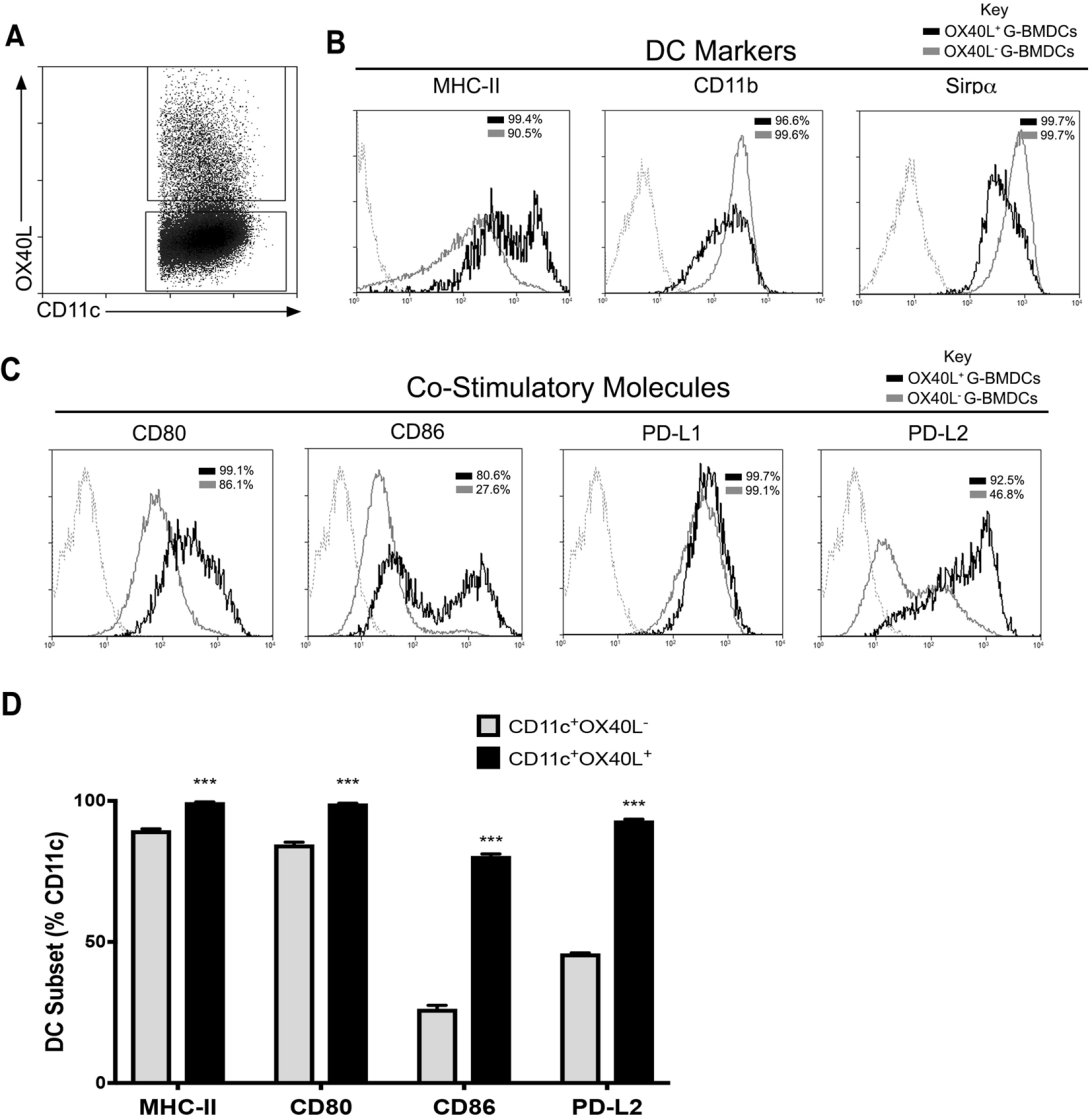
Costimulatory and co-inhibitory molecules are highly expressed on OX40L^+^ G-BMDCs. (**A**) Representative dot plot indicating the subdivided populations OX40L^+^ BMDCs (top quadrant) and CD11c^+^OX40L^−^ (bottom quadrant). (**B**,**C**) Representative histograms showing surface expression of the indicated markers by OX40L^+^ G-BMDCs (black) and OX40L^−^ G-BMDCs (grey) subsets. Dotted line represent stained controls. (**D**) Bar graphs showing the percent expression of indicated markers on OX40L^−^ G-BMDCs (grey) and OX40L^+^ G-BMDCs (black) subsets. Values are expressed as means ± SEM (n = 3; **p < 0.01, ***p < 0.001).

To more thoroughly characterize the cells differentiated by GM-CSF and to assess the potential functional differences between OX40L^+^ and OX40L^−^ G-BMDCs, we analyzed the expression of various co-stimulatory/co-inhibitory markers as these molecules have been implicated in the regulation of Tregs. Cells were first identified by their expression of OX40L or lack thereof on CD11c expressing cells (Fig. 2A). We then identified increased expression of MHC-II with high expression of the myeloid marker, CD11b, and Sirpα on OX40L^+^ G-BMDCs (Fig. 2B). Sirpα expression has been shown to be inversely correlated with CD8α expression suggesting G-BMDCs are of CD11c^+^CD8α^−^ origin^45^. DC-restricted deficiency of CD80 and CD86 has been shown to lead to a reduction in peripheral Treg frequencies implicating a role for CD80/CD86 in peripheral Treg homeostasis^46^. We found OX40L^+^ G-BMDCs to express higher levels of the co-stimulatory marker CD80, but significantly increased levels of CD86 compared to OX40L^−^ DCs. PDL2 has been implicated to play a critical role in immune tolerance by negatively regulating T-cell immune responses^47^. Our assessment revealed OX40L^+^ G-BMDCs to express significantly higher levels of PDL2 when compared to OX40L^−^ counterparts (Fig. 2C,D). These results suggest that OX40L^+^ G-BMDCs highly express co-stimulatory molecules involved in DC Treg homeostasis and can be identified by MHCII, CD11b, Sirpα, CD80, CD86, and PDL2 expression.

### GM-CSF administration increases functionally suppressive Tregs in the periphery

**Figure 3:**
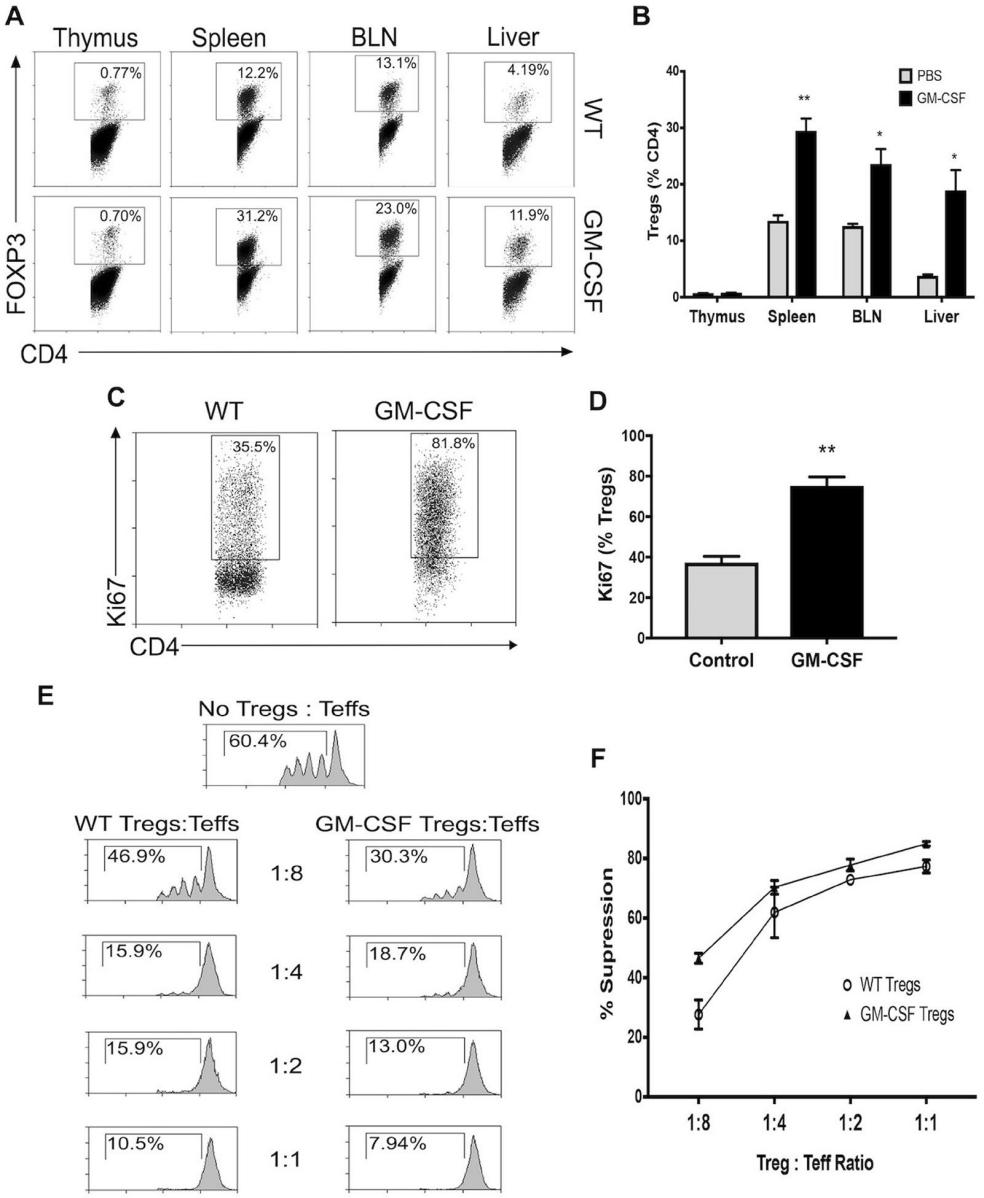
GM-CSF administration increases functionally suppressive Tregs in peripheral lymphoid organs. GM-CSF was administered to C57BL/6 mice daily for 4 days (n = 3). (**A**) Representative dot plots of percent Tregs within various tissues of untreated and GM-CSF treated mice. (**B**) Bar graphs showing the percent Foxp3^+^ Tregs in indicated tissues from untreated and GM-CSF treated mice. Values are expressed as means ± SEM (n = 3; *p < 0.05, **p < 0.01). (**C**) Representative dot plot of percent Tregs with Ki67 expression from the spleen of untreated and GM-CSF treated mice. (**D**) Summary bar graphs for data shown in C Values are expressed as means ± SEM (n = 3; **p < 0.01). (**E**) Treg suppression analysis was performed on Tregs isolated from the spleens of untreated and GM-CSF mice. Representative histograms are shown. (**F**) Percent suppression was determined at each ratio of Tregs/Teff. Values are expressed as means ± SEM (n = 3; n.s. = not significant).

Since we had previously observed Treg expansion in *ex vivo* co-cultures of CD4 T-cells with OX40L^+^ G-BMDCs, we attempted to replicate *ex vivo* conditions by administering short-term treatment of GM-CSF to age- and sex-matched C57BL/6 mice. Treg analysis of thymic tissue revealed no differences in Treg frequencies, however, a significant increase in the percentage of Tregs was observed in the spleen, BLN and the liver of GM-CSF treated mice (Fig. 3A,B). Additionally, in order to determine whether increased Treg frequencies is due to enhanced proliferation, we assessed the expression of the cell cycle proliferation marker Ki67 by intracellular staining. After 4 days of GM-CSF treatment, the proportion of proliferating Tregs (Ki67^+^ Tregs) nearly doubled in the spleen of GM-CSF treated mice compared to WT controls (Fig. 3C,D). To further confirm that increase in Tregs numbers seen in GM-CSF treated mice was due to proliferation, and not increased survival, we analyzed the expression of pro-survival factor BCL2 and proliferation marker Ki67 in GM-SCF expanded Tregs. BCL2 is an anti-apoptotic factor that promotes cell cycle arrest and ensures cell survival, while a reduction in its levels could lead to cell proliferation^48–50^. We observed reduced BCL2 expression and increased Ki67 expression in GM-CSF expanded Tregs compared to WT-Tregs (Supplementary Fig. S1). Thus, the increase in the proportion of Tregs in GM-CSF treated mice is likely due to proliferation of Treg cells rather than increased survival.

Furthermore, we assessed the suppressive capacity of the Tregs isolated from control and GM-CSF treated mice to determine whether the suppressive function of Tregs was diminished due to their expansion. Tregs isolated from GM-CSF treated mice suppressed the proliferation of stimulated T-effector cells at similar levels as WT Tregs (Fig. 3E). The percent T-effector suppression was not significantly different at any dilution of Tregs to T-effectors between WT Tregs and Tregs from GM-CSF treated mice (Fig. 3F), suggesting Treg suppression was not impaired or altered with GM-CSF treatment or upon undergoing proliferation. Collectively, these results suggest GM-CSF induced the proliferation of functionally suppressive Tregs in the periphery.

### GM-CSF induces OX40L^+^ DCs that can expand Tregs

**Figure 4:**
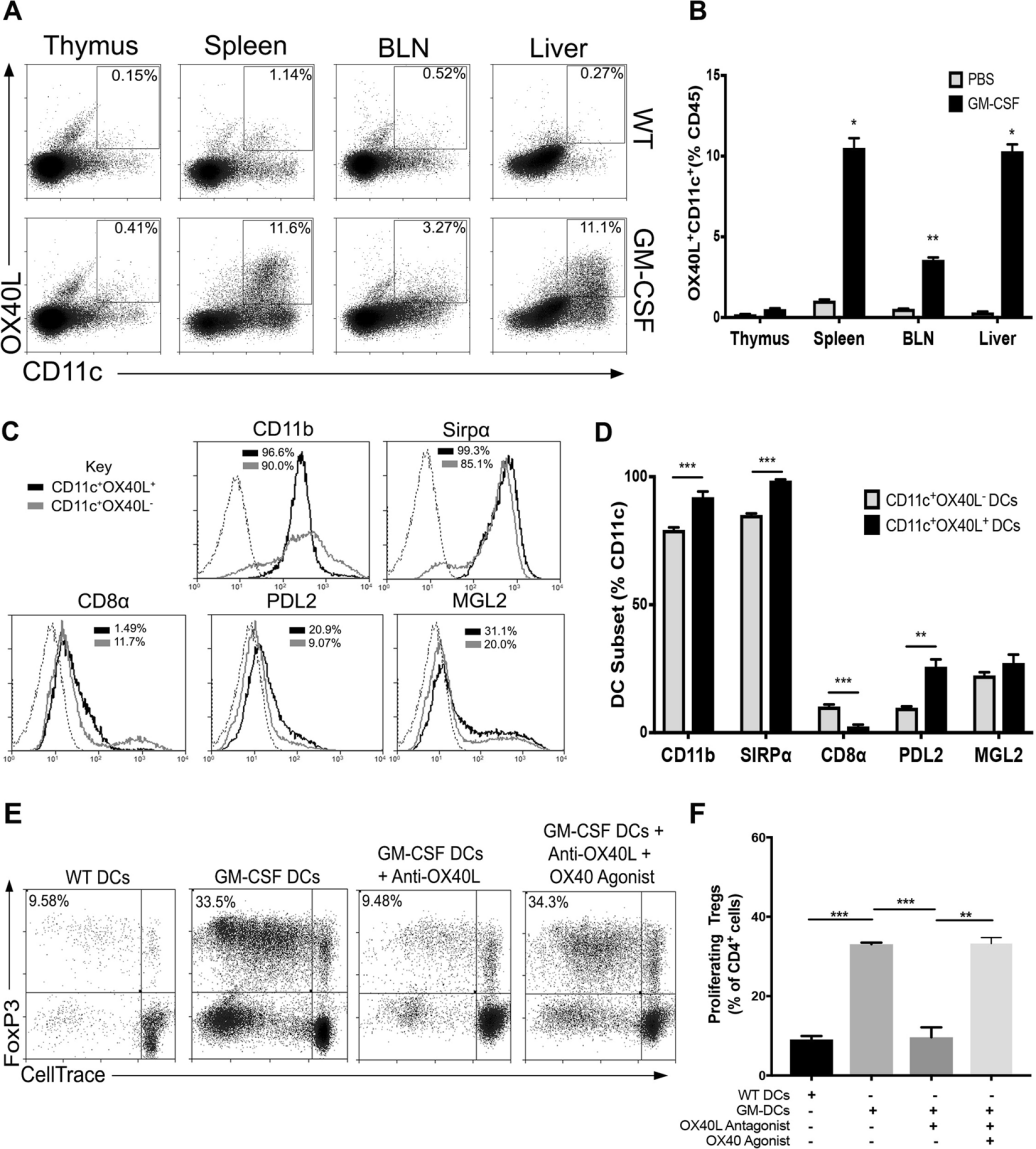
*In vivo* derived DCs expand Tregs through OX40L. (**A**) Representative dot plots of OX40L^+^CD11c^+^ expression within various tissues of untreated and GM-CSF treated mice (n = 3). (**B**) Bar graphs show percent OX40L^+^CD11c^+^ DCs in indicated tissues. Values are expressed as means ± SEM (n = 3; *p < 0.05, **p < 0.01). (**C**) Representative histograms of indicated markers on OX40L^+^CD11c^+^ (black) compared to OX40L^+^CD11c^−^ (grey) isolated cells. Dotted lines represent stained controls. (**D**) Bar graphs showing the expression of indicated markers on OX40L^+^CD11c^+^ DCs and OX40L^−^CD11c^+^ DCs. Values are expressed as means ± SEM (n = 3; **p < 0.01, ***p < 0.001). (**E**) CD11c^+^ DCs were isolated from the spleens of GM-CSF treated or WT mice and co-cultured with CD4 T-cells with or without OX40L blockade and/or supplemented with OX40 agonist. Representative dot plots are shown. (**F**) Summary bar graphs showing percent proliferating Foxp3^+^ Tregs in indicated co-cultures. Values are expressed as means ± SEM (n = 3; **p < 0.01, ***p < 0.001).

Since we observed a pronounced increase in Tregs upon GM-CSF administration, we investigated whether GM-CSF induced the generation of OX40L^+^ DCs, to determine whether this OX40L^+^ DC subset was responsible for the observed *in vivo* Treg expansion. As expected, we did not find an observable amount of OX40L^+^ DCs in the tissues of WT animals (Fig. 4A), as others have indicated OX40L expression to be observed in inflammatory settings^51^. Upon GM-CSF treatment, we also did not observe an increase of OX40L^+^ DCs in the thymus, which corresponded with the lack of Treg increase in this tissue. We did, however, observe significant increases of OX40L^+^ DCs in the spleen, brachial lymph nodes, and the liver of GM-CSF treated mice (Fig. 4A,B); tissues where we had previously observed significant increases in the percent Treg population upon GM-CSF treatment. These results suggested that OX40L^+^ DCs may be involved in the increase of peripheral Tregs found upon GM-CSF administration.

Further phenotypic characterization of *in vivo* derived splenic OX40L^+^ DCs identified phenotypic similarities to *ex vivo* derived OX40L^+^ G-BMDCs. CD11b and Sirpα were highly expressed on *in vivo* derived OX40L^+^CD11c^+^ (Fig. 4C,B) which we had seen previously on OX40L^+^ G-BMDCs. Furthermore, CD8α^−^ expression was found to be significantly reduced on OX40L^+^CD11c^+^ which we predicted as Sirpα expression is inversely correlated with this molecule^45^. Additionally, PDL2, a surface molecule we found to be significantly upregulated on OX40L^+^ G-BMDCs compared to OX40L^−^ G-BMDC controls, was also found to be upregulated on *in vivo* derived OX40L^+^ DCs compared to OX40L^−^ DCs (Fig. 4C,D). Furthermore, we investigated the expression of another molecule MGL2 on OX40L^+^ DCs. The molecules PDL2 and MGL2 have been recently found on a DC subset identified in a tumor-induced GM-CSF microenvironment with the capability of suppressing CD8^+^ T-cells and expanding Tregs, respectively^52^. Similarly, on our splenic OX40L^+^ DCs isolated from GM-CSF treated mice, expression of MGL2 was observed (Fig. 4C,D). These results suggest that *in vivo* derived OX40L^+^ DCs share phenotypic similarities with *ex vivo* derived OX40L^+^ G-BMDCs and, further, may point to undiscovered mechanisms of immune tolerance through PDL2 signaling.

Although we have shown OX40L^+^ DCs to co-localize to tissues that exhibited increased Treg frequencies and OX40L^+^ DCs shared similar phenotypic characteristics to OX40L^+^ G-BMDCs, it was important to determine whether *in vivo* derived OX40L^+^CD11c^+^ DCs possessed the capacity to expand Tregs, much like the function of *ex vivo* derived OX40L^+^ G-BMDCs. We assessed the function of Treg expansion in CD4^+^ T-cell co-cultures with CD11c^+^ cells isolated from the spleens of GM-CSF or WT treated mice. In comparison to cultures with WT CD11c^+^ DCs, CD4^+^ T-cells co-cultured with splenic CD11c^+^ DCs isolated from GM-CSF treated mice showed a significant increase in the proportion of proliferating Tregs (Fig. 4E,F). Furthermore, to determine the role of OX40L^+^ on CD11c^+^ DCs in this Treg expansion, we utilized an OX40L antagonist. Upon addition of the OX40L antagonist to the CD4^+^ T-cell: CD11c^+^ DC co-culture, the percentage of proliferating Tregs in the co-culture was significantly reduced (Fig. 4E,F). However, upon reconstitution with an OX40 agonist the percent Treg expansion was restored to previous levels (Fig. 4E,F). These results showed that *in vivo* generated OX40L^+^CD11c^+^ DCs share similar phenotype and function with OX40L^+^G-BMDCs and were likely the DC involved in the expansion of peripheral Tregs in GM-CSF treated mice.

### OX40L^+^ G-BMDCs may represent a non-steady state DC subset

**Figure 5:**
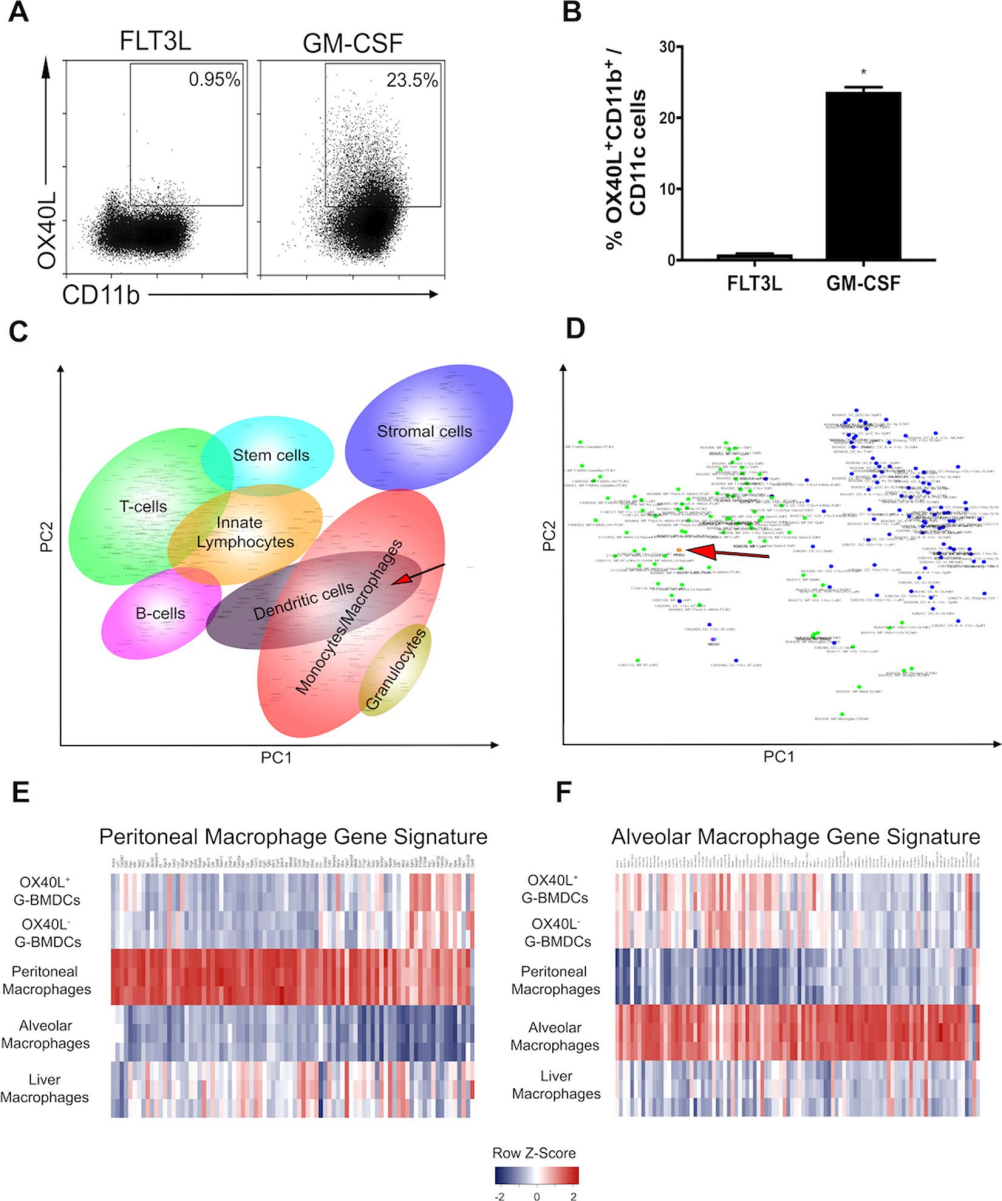
OX40L^+^ CD11c^+^ DCs represent non-steady state identity. (**A**) Representative dot plots of bone marrow precursor cells cultured in FLT3L or GM-CSF and assessed for the differentiation of OX40L^+^ G-BMDCs. Gated on CD11c^+^ cells. (**B**) Bar graphs showing the percent expression of OX40L^+^ G-BMDCs in indicated BM cytokine cultures. Values are expressed as means ± SEM (n = 3; *p < 0.05). (**C**) Principal-component analysis plot of all genes expressed in immune cell types from the ImmGen database and CD11c^+^OX40L^+^ G-BMDCs. Each dot represents cell type sample. Red arrow represents CD11c^+^OX40L^+^ G-BMDCs. (**D**) Subsequent principal component analysis of all genes expressed in macrophage and dendritic cell subtypes from the ImmGen database compared to CD11c^+^OX40L^+^ G-BMDCs. Each dot represents cell type sample, green dots are macrophages subtypes and blue dots are dendritic cell subtypes. Red arrow represents CD11c^+^OX40L^+^ G-BMDCs. (**E**,**F**) Heatmap of alveolar and peritoneal gene signature in CD11c^+^OX40L^+^ G-BMDCs compared to CD11c^+^OX40L^−^ G-BMDCs, alveolar macrophages, liver macrophages, and peritoneal macrophages from the ImmGen database.

To determine whether the induction of OX40L on CD11c^+^ is a specific property of GM-CSF, we assessed the functional capacity of GM-CSF and FLT3L, two cytokines strongly linked to DC development, to differentiate OX40L^+^ BMDCs from BM precursor cells (Fig. 5A,B). BM precursor cells cultured with FLT3L failed to differentiate OX40L^+^ BMDCs. In contrast, OX40L^+^ G-BMDCs were abundant in GM-CSF BM cultures suggesting the induction of OX40L^+^ BMDCs is unique property of the GM-CSF cytokine (Fig. 5A,B).

There has been much debate over the role of GM-CSF in DC development^53^. GM-CSF has been postulated to play a role in inflammatory processes rather than under steady-state conditions due to the lack of alterations of hematopoietic populations in GM-CSF receptor-deficient mice^54^. This notion is further supported by evidence of GM-CSF levels being typically very low or absent in steady-state conditions; however, upon exposure to strong inflammatory stimuli GM-CSF, production is rapidly increased^55^. To determine whether OX40L^+^ DCs represent a non-steady state immune cell, we performed a principal component analysis (PCA) comparing the transcriptome of OX40L^+^ G-BMDCs to that of all myeloid and lymphoid lineages in the Immgen database. The ImmGen Project is a collaboration between a group of immunologists and computational biologists who seek to collect and generate comprehensive gene expression data to molecularly characterize the immune system of the mouse^56^. The principal-component analysis (PCA) of the expression of all genes revealed OX40L^+^ G-BMDCs clustered closely with dendritic cell and macrophage populations (Fig. 5C). When this PCA was restricted to only the transcriptomes of macrophages and dendritic cells, OX40L^+^ G-BMDCs more closely clustered with the macrophage population with the closest relatives being alveolar macrophages, peritoneal macrophages and liver macrophages (Fig. 5D). Consistent with these results, OX40L^+^ G-BMDCs expressed only some of the core signature genes of alveolar and peritoneal macrophages, suggesting that while OX40L^+^ G-BMDCs cells may have a similar transcriptome to those of alveolar and peritoneal macrophages, they are distinct from the steady-state cells of the immune system (Fig. 5E,F).

### OX40L^+^ G-BMDCs express a unique transcriptional profile

**Figure 6:**
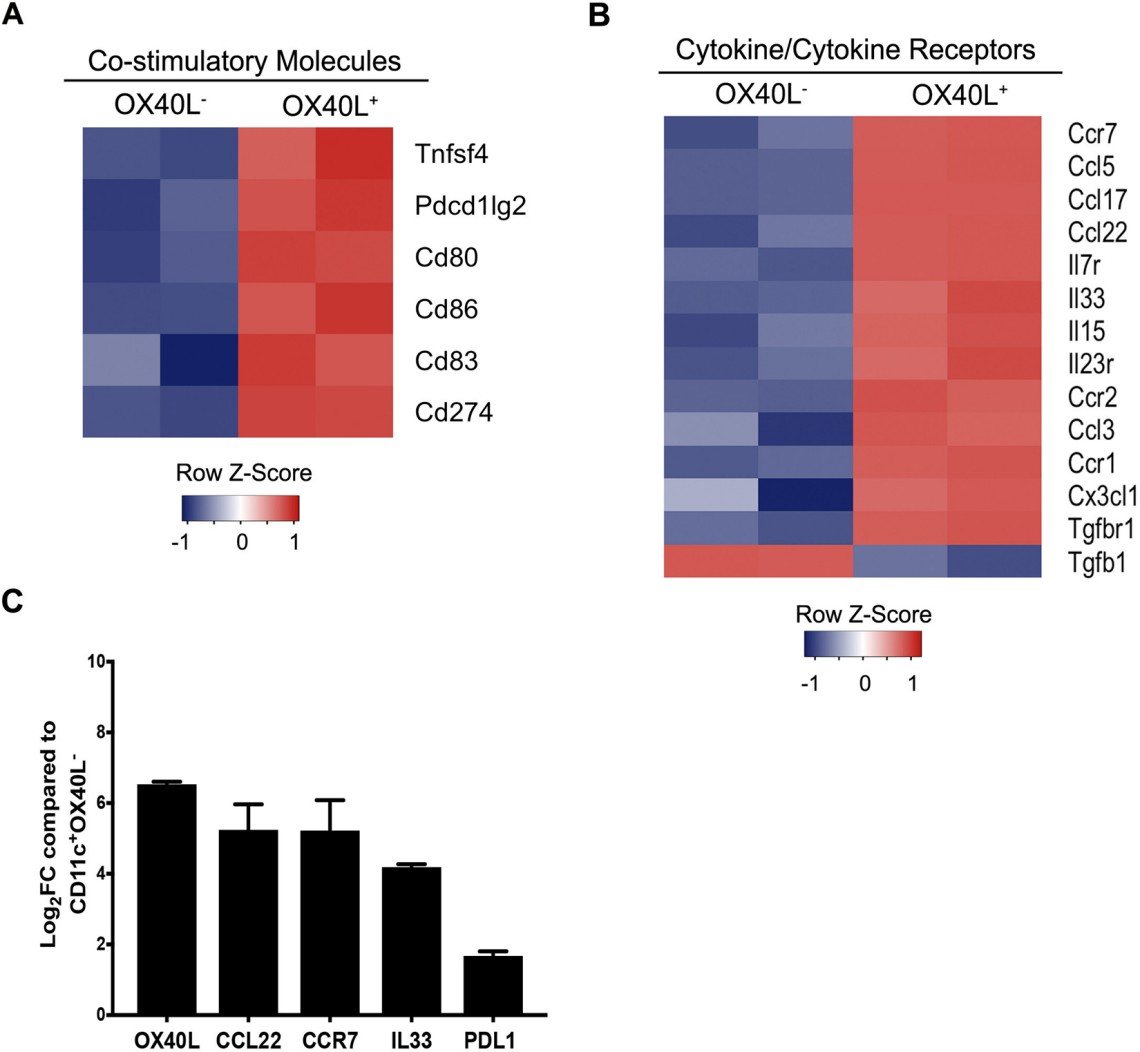
Microarray analysis reveals differential expression between OX40L^+^ G-BMDCs and OX40L^−^ G-BMDCs. (**A**,**B**) Heatmap of gene expression for select indicated categories of genes comparing sorted OX40L^+^ G-BMDCs to OX40L^−^ G-BMDCs. Individual replicates are shown. (Fold change > 2, p-value < 0.05) (**C**) RT-qPCR of selected genes. Fold changes (Log_2_FC) are shown relative to OX40L^−^ G-BMDCs. Values are expressed as means ± SEM (n = 4).

Contrary to other reports, our results suggest OX40L^+^ DCs may play a preventative role in autoimmunity through the expansion of Tregs. To further understand the molecular signature of OX40L^+^ G-BMDCs, and thus the tolerogenic mechanism implemented by OX40L^+^ G-BMDCs, we conducted a genome-wide microarray analysis of mRNA expression between OX40L^+^ and OX40L^−^ G-BMDCs to identify molecules that may lead to the functional differences observed between these two DC populations. Microarray analysis revealed vast differences in transcriptome phenotype of OX40L^+^ G-BMDCs compared to OX40L^−^ G-BMDCs. Confirming the phenotype of these cells, *OX40L* (*Tnfsf4)* was the highest differentially expressed costimulatory gene with a 6-fold increase in expression in OX40L^+^ G-BMDCs (Fig. 6A,C). As we saw previously from the flow cytometry results, the OX40L^+^ G-BMDC population presented a stronger co-stimulatory phenotype with significantly increased expression of *CD86*, as well as the co-inhibitory molecule *PDL2* (Fig. 6A). Additionally, *CCR7*, a chemokine receptor involved in the trafficking of Tregs to lymphoid sites and implicated in the maturation and mobilization of dendritic cells, was found to be the most significantly upregulated in the OX40L^+^ G-BMDCs (Fig. 6B). The lack of CCR7 has been implicated in the manifestation of spontaneous autoimmunity^57^. Furthermore, among the cytokines, IL-33, a cytokine highly implicated in the generation of Foxp3^+^ Tregs^58,59^ was the most differentially expressed cytokine with higher expression levels in OX40L^+^ G-BMDCs compared to OX40L^−^ G-BMDCs (Fig. 6B). *IL7R* was the most differentially expressed among the interleukin receptor family (Fig. 6B). *IL7R* deficiencies have been shown to contribute to autoimmune diseases such as SCID^60^. Furthermore, CCL5, *CCL17*, and *CCL22*, chemokine ligands shown to be involved in the trafficking of Tregs to inflammatory sites^61,62^, were also among the chemokine ligands significantly upregulated (Fig. 6B). In this regard, it is interesting to note that we have observed increased expression of CCL17/CCL22 chemokine receptor CCR4 in proliferating Tregs relative to resting Tregs from G-BMDC co-cultures in our previous microarray study (GEO data base accession No. GSE81051)^63^ suggestive of recruitment of these Tregs to inflammatory sites. RT-qPCR confirmed the differential expression *OX40L*, *CCL22*, *CCR7*, and *PDL1* with increased transcriptional expression compared to OX40L^−^ G-BMDCs (Fig. 6C). The results of this microarray analysis identifies differentially expressed molecules that may play important roles in the function of the novel OX40L^+^ DC subset and further suggests a tolerogenic role of OX40L^+^ DCs in physiology. Taken all together, our study suggests that OX40L^+^ G-BMDCs can be induced *in vivo* upon GM-CSF administration and may represent a non-steady state DC subtype involved in Treg homeostasis and immunosuppression.

## Discussion

In this study, we determined that *in vivo* derived OX40L^+^CD11c^+^ DCs induced upon GM-CSF treatment are phenotypically and functionally equivalent to *ex vivo* derived OX40L^+^ G-BMDCs. There has been debate over the role of GM-CSF in normal DC development. GM-CSF has been postulated to play a role in inflammatory processes rather than under steady state. This contention is supported by the lack of alterations of hematopoietic populations in GM-CSF receptor deficient mice with minor alterations in alveolar macrophages^54^. Under steady state, GM-CSF levels are very low or absent; however, upon exposure to strong inflammatory stimuli GM-CSF production is rapidly increased^55^ which in turn increases the number of inflammatory DCs. In contrast, there is a growing body of evidence that GM-CSF can protect animals from developing autoimmune diseases through the expansion of “tolerogenic” DCs^9,11–13^. Additional, studies from our laboratory showed that BMDCs, derived from the bone marrow of either WT or MHC Class-II^−/−^ mice differentiated *ex vivo* in the presence of GM-CSF (G-BMDCs), could selectively expand Tregs independent of TCR signaling but was critically dependent on the expression of OX40L on DC surface^15,16^. Supporting this data, in this study, we have demonstrated that sorted OX40L^+^ G-BMDCs were responsible for the expansion of Tregs. Furthermore, using Ki67 vs BCL2 staining we confirmed that increased proliferation, but not survival, is the likely principle mechanism underlying increased Tregs seen in GM-CSF treated mice. However, secondary mechanisms such as Treg mobilization might also contribute to increased Tregs upon GM-CSF treatment as suggested by increased chemokine CCL17 and CCL22 expression in OX40L+DCs and chemokine receptor CCR4 expression as we have earlier noted in expanded Tregs. More importantly, expanded Tregs not only expressed suppressive phenotypic markers such as CTLA4, CD39 and Helios, but were functionally competent.

Furthermore, we have previously shown, upon treatment with GM-CSF in mice, a significant increase in Tregs with a corresponding increase in the CD11c^+^CD8a^−^ DC subset^11^. We suggested that the CD11c^+^CD8α^−^ DC subset may play a role in the amelioration of autoimmune diseases through Treg expansion and increased IL-10 production. Based on our current findings, we suggest that a subset of the CD11c^+^CD8α^−^ DCs express OX40L and this subset may be the critical “tolerogenic” DC subtype capable of selectively expanding Tregs and thus suppressing autoimmunity. CD8α^−^ DCs in *ex vivo* derived co-cultures have previously been identified by high levels of expression of the molecule Sirpα^45^. In this regard, we found OX40L^+^ G-BMDCs to express higher levels of Sirpα, thus identifying OX40L^+^ G-BMDCs as a subset of CD11c^+^CD8α^−^ DCs. In addition to Sirpα expression, we evaluated the co-stimulatory and co-inhibitory markers of OX40L^+^ G-BMDCs compared to OX40L^−^ G-BMDCs to reveal molecules that may play a role in the functional differences between the two subsets. OX40L^+^ G-BMDCs expressed significantly increased levels of the co-stimulatory molecule CD86, as well as the co-inhibitory molecule PDL2. Selective reduction of CD80 and CD86 on CD11c^+^ DCs in mice has been shown to significantly reduce the frequencies of peripheral Tregs suggesting a role for CD80/CD86 in Treg homeostasis^46^. Furthermore, PDL2 has been implicated in negatively regulating T-cell immune responses and thus, promoting tolerance^47^. Additionally, of the most upregulated genes, OX40L^+^ G-BMDCs exhibited increased expression of molecules involved in Treg expansion (IL-33)^58,59^, Treg recruitment (CCL5, CCL22, and CCL17)^61,62^, and DC maturation (CD80, CD86, CCR7)^57^, further confirming the tolerogenic immune phenotype of OX40L^+^ G-BMDCs.

Complicating this paradigm, however, are reports suggesting that GM-CSF differentiated bone marrow cultures may yield discrete *in vitro* generated macrophages and dendritic cells that may not correspond to immune cell types found *in vivo*^36^. However, our current studies show that treatment with GM-CSF increased Treg populations in the spleen, brachial lymph nodes and the liver of treated mice. As suspected, an investigation of OX40L expression on CD11c^+^ revealed OX40L to be negligibly expressed in WT mice. However, upon GM-CSF treatment OX40L expression on CD11c^+^ was significantly increased in the spleen, BLN and the liver of treated mice, but not in the thymus. Tissue specific increases in OX40L^+^ on CD11c^+^ correlated with increases in Treg populations, without causing loss of their suppressive function, in the same tissues upon GM-CSF treatment, suggesting a role for OX40L^+^CD11c^+^ DCs in this peripheral Treg expansion.

OX40L can act as a co-stimulatory signal for TCR-dependent effector T-cell activation under pro-inflammatory conditions^64^. Physiologically, OX40L^+^ expression appears to be restricted to inflammatory sites and is absent in wild-type, uninfected mice. For example, OX40L^+^ DCs have been described in the pancreas and secondary lymphoid organs of 11–13 week-old pre-diabetic NOD mice^26^, in the inflamed kidneys of Lupus patients^27^, and in EAE brain tissues^65^. Furthermore, based on the outcome of studies using agonistic OX40 signaling or OX40L blockade, it has been postulated that OX40L^+^ DCs were the cause of inflammation and autoimmune disease. However, OX40L has been shown to be induced on various immune cells such as B-cells^20^, macrophages^21^, mast cells^22,23^ and natural killer cells^24^ and therefore, systemic administration of OX40L and blockade of OX40 may have broader effects, and thus differ mechanistically from the way in which OX40L^+^ DCs specifically exert their tolerogenic effect through Treg expansion. Additionally, OX40L signaling has been reported to inhibit the Foxp3 expression and Treg functions in other studies under pro-inflammatory conditions^66,67^ and also shown to inhibit extra-thymic TGF-β induced Treg (iTreg) differentiation from Tconv cells^68^, a process requiring TCR activation. In contrast, earlier we have shown that OX40 signaling can promote thymic Treg differentiation in the TCR-independent phase^50^. Interestingly, that the majority of Tregs expanded, in a TCR-independent manner, in the presence of OX40L+DCs were Helios+ natural Tregs (nTregs) (differentiated in the thymus). We and others have observed that treatment of NOD mice with OX40L or OX40 agonist was protective when given at 6–8 weeks of age, and this protective effect was mediated through expansion of Tregs^69–71^. However, OX40L treatment of NOD mice during effector phase of diabetes around 10–12 weeks of age exacerbated the disease^26,69^. Moreover, co-treatment of NOD mice with OX40L with immune modulators like Jagged-1 and IL-2 expanded functional Tregs and protected against T1D^63^ and graft rejection^33^. Taken together, it is clear that the OX40L may exert divergent effects on different cell types based on the local cytokine milieu and the presence or absence of TCR signaling.

Principal component analysis using gene expression data from OX40L^+^ DCs and cell subsets from the ImmGen database revealed OX40L^+^ DCs to closely cluster with alveolar, peritoneal, and liver macrophages. Although, we found similarity between the transcriptional profile of OX40L^+^ DCs and the gene signatures of alveolar and peritoneal macrophages the OX40L^+^ DC gene signature remained distinct from those of the other cells. Since the ImmGen database contains transcriptional data acquired from steady-state, non-infected mice, our data suggest that OX40L^+^ DCs could represent a non-steady state dendritic cell subtype induced under inflammatory conditions through the increased production of GM-CSF that could play a regulatory feedback role to temper inflammation.

GM-CSF derived DCs have long been utilized in therapeutic vaccines for cancer. The efficacy, however, of these DC tumor vaccines has been called into question^72^. While many DC vaccines have exhibited antigen specific immune responses, clinical responses have been low^72^. Based on our findings, it could be hypothesized that OX40L^+^ G-BMDCs, when used for DC-based vaccination, may suppress anti-tumor effector immune responses through the expansion of Tregs, and therefore, lead to suboptimal immune response and/or treatment failure. In fact, in a melanoma DC vaccine clinical trial with low-dose cytokines (IL-2 and IFN-gamma), Tregs were significantly increased by the fourth dose of the DC vaccine and were correlated with disease progression^73^. These findings combined with our current results suggest that it is prudent to consider the possibility of inducing tolerogenic DCs, such as OX40L^+^ G-BMDCs, by GM-CSF, while developing/optimizing therapeutic tumor DC vaccinations.

Additionally, the tolerogenic properties of OX40L^+^ DCs could also play a critical role in metastatic tumor microenvironments. PDL2 and MGL2 have been identified on CD11c^+^CD11b^+^ cells in a metastatic tumor-released GM-CSF microenvironment and implicated in the suppression of CD8 T-cells and the expansion of Tregs, respectively^52^. In many cancers, poor outcomes are associated with increased Treg frequencies and low CD8 T-cell infiltration^74,75^. Depletion of this DC subset was found to enhance tumor immunity and inhibit tumor metastasis^52^. In our studies, we detected expression of MGL2, along with the upregulation of the co-inhibitory molecule PDL2 on *in vivo* derived OX40L^+^ DCs from GM-CSF treated mice. It is possible that OX40L^+^ DCs may play another not yet discovered role in the suppression of CD8 T-cells through PDL2 signaling. Therefore, further investigation into the development and function of OX40L^+^ DCs could yield tumor-specific mechanisms in the treatment of metastatic cancers.

In conclusion, our results suggest OX40L^+^ DCs induced *in vivo* by GM-CSF play a role in the expansion of functional Tregs as a mechanism of maintaining immune homeostasis during inflammation or infection. The role of OX40L^+^ DCs should be further explored as they could be potentially manipulated for optimal therapeutic use in the treatment of autoimmune disease, cancer, or DC-based vaccine development.

## Materials and Methods

### Animals

C57BL/6J female mice (6- to 12-week-old) were purchased from the Jackson Laboratory (Bar Harbor, ME, USA). Mice were housed in the Biological Resources Laboratory Facility at the University of Illinois (Chicago, IL, USA) and provided food and water ad libitum. All animal experiments were approved and performed in accordance with the guidelines set forth by the University of Illinois at Chicago Animal Care and Use Committee.

### Reagents

Recombinant mouse GM-CSF was purchased from Miltenyi Biotec (Auburn, CA). Recombinant mouse FLT3L was purchased from Gemini Bio-Products (West Sacramento, CA). CellTrace Violet cell proliferation kit was purchased from ThermoFisher Scientific (Waltham, MA). Anti-FOXP3, anti-CD4, anti-OX40L, anti-CD11c, anti-Helios, anti-CTLA4, anti-CD39, anti-CD11b, anti-Sirpα, anti-CD80, anti-CD86, anti-CD274, anti-CD273, anti-MHCII, anti-Ki67, and anti-MGL2 fluorescently coupled antibodies were purchased from ThermoFisher Scientific (Waltham, MA). Foxp3/Transcription Factor Staining Buffer Kit was purchased from Tonbo Biosciences (San Diego, CA). Purified anti-OX40L (RML134L) was purchased from Biolegend (San Diego, CA) and purified anti-OX40 agonist (OX-86) was purchased from ThermoFisher Scientific (Waltham, MA).

### Flow Cytometry Analysis

For flow cytometry analysis, cells were washed with PBS containing 0.5% BSA. For surface staining, the cells were labeled with specified FITC-, PE-, EFluor® 450-, APC-, and APC-Cy7- conjugated monoclonal antibodies for 30 min. For cell proliferation assays, the cells were labeled with CellTrace Violet, fixed, permeabilized, and incubated with fluorescent coupled antibodies for intracellular staining. Stained cells were washed and analyzed by CyAn ADP Analyzer (Beckman Coulter) and data analysis was performed using Summit v4.3 software (Beckman Coulter).

### Isolation of DC and T-cell populations

Bone marrow cells were cultured in complete RPMI medium containing 10% heat-inactivated FBS in the presence of 20 ng/ml GM-CSF. Fresh medium containing 20 ng/mL GM-CSF was added on days 3 and 6. On the 8th day, non-adherent CD11c^+^ DCs (G-BMDCs) or specific subpopulations of G-BMDCs (i.e., OX40L^+^ or OX40L^−^) were sorted using a MoFlo flow cytometer (Beckman/Coulter) following staining with appropriate antibodies. CD4^+^ cells were isolated from the spleens by using the Mouse CD4^+^ T Cell Isolation Kit II from Miltenyi Biotec (San Diego, CA).

### *In vitro* DC and T-cell co-cultures

DC-T-cell co-culture experiments were conducted in triplicate with isolated T-cells and either G-BMDCs or isolated DC populations, pooled from three mice. G-BMDCs (5 × 10^4^) or OX40L^+^CD11c^+^ DCs (5 × 10^4^) were cultured with CD4^+^ T-cells (1 × 10^5^) at a ratio of 1:2 for 5 days. For proliferation assays, CD4^+^ T-cells were labeled with CellTrace Violet (Life Technologies) according to manufacturer’s protocol. Some cultures were supplemented with an anti-OX40L (10 μg/ml) and/or an OX40 agonist (OX86; 10 μg/ml).

### GM-CSF treatment

Amine-directed PEGylation of the recombinant mouse GM-CSF was performed following the protocol as detailed previously^76^. Age and sex-matched C57BL/6J mice were treated daily for 4 days with 5 ug/mouse of pegylated GM-CSF intraperitoneally or with PBS as a vehicle control. 24 hours after the last treatment, mice were sacrificed, and organs were harvested for flow cytometric analysis.

### Treg Suppression Assay

CD4^+^CD25^+^ splenic Tregs were sorted from control and GM-CSF treated mice and were co-cultured with CellTrace-Violet labeled, anti-CD3/anti-CD28 microbead stimulated CD4^+^CD25^−^ Teff cells at 1:8, 1:4, 1:2, 1:1 ratios. Proliferation was measured by the dilution of CellTrace-Violet and the percentage of Teff cell proliferation suppression was calculated.

### Microarray and Gene Expression analysis

OX40L^+^ and OX40L^−^ G-BMDCs were sorted by fluorescence-activated cell sorting and total RNA was extracted using RNAeasy Mini isolated kit (Qiagen). For each population, mRNAs were profiled on Affymetrix MoGene-1_0-st-v1 expression microarrays. Labeling, hybridization, and staining of microarrays were performed according to the manufacturer’s protocol. Data was analyzed using Bioconductor 3.4 (http://ww.bioconductor.org) running on R 3.3.2 (http://www.R-project.org). Raw data was preprocessed by RMA normalization using the R Bioconductor “oligo” package^77^. Top differentially expressed genes between two OX40L positive and two OX40L negative samples were obtained using the Bioconductor “limma” package^78^ using a false-discovery rate (FDR) of less than 0.05 and absolute fold change greater than 2.0 as thresholds. Heat maps were created by calculating row Z-score. A principal component analysis (PCA) was performed on two microarray datasets with the same Affymetrix platform from the Immunological Genome Project (ImmGen)^56^: GSE15907 and GSE37448, along with our samples. Raw datasets were preprocessed by RMA normalization. DC and macrophage signature genes were obtained from Gautier *et al*., 2012 and Miller *et al*., 2012^79,80^.

### RT-PCR

Total RNA was extracted using TRIzol reagent (Invitrogen) following manufacturer’s instructions. The cDNA synthesized from total RNA was used for RT-qPCR analysis with Fast SYBR green master mix (Applied Biosystems) and gene-specific primers (listed in Supplementary Table 1) by using AB ViiA7 RT-qPCR instrument (Applied Biosystems). Gene expression values were calculated by comparative ∆Ct method after normalization to 18 s internal control and expressed as log fold change over respective controls. The following primer sets were used to amplify the indicated products: OX40L-F: AATCTGGAAAACGGATCAAGGC; OX40L-R: CAGGCAGACATAGATGAAGCAC; PDL1-F: GCTCCAAAGGACTTGTACGTG; PDL1-R: TGATCTGAAGGGCAGCATTTC; CCR7-F: TGTACGAGTCGGTGTGCTTC, CCR7-R: GGTAGGTATCCGTCATGGTCTTG; IL33-F: TCCAACTCCAAGATTTCCCCG; IL33-R: CATGCAGTAGACATGGCAGAA, CCL22-F: AGGTCCCTATGGTGCCAATGT; CCL22-R: CGGCAGGATTTTGAGGTCCA, 18 s rRNA-F: GATCCATTGGAGGGCAAGTCT; 18 s rRNA-R: CCAAGATCCAACTACGAGCTTTTT.

### Statistical analysis

Statistical analyses were performed using Prism GraphPad (V7.0). Data were expressed as Mean ± SEM of multiple experiments. Student’s two tailed t-test was used to compare two groups, whereas ANOVA with Tukey’s multiple comparisons was used to compare more than two groups. A p-value < 0.05 was considered significant. * indicates p < 0.05; ** indicates p < 0.01; *** indicates p < 0.001.

## Electronic supplementary material

Supplementary Dataset 1
