# Supplementary material for: Identification of a Novel OX40L+ Dendritic Cell Subset That Selectively Expands Regulatory T cells
Source: Sci Rep. 2018 Oct 8;8:14940. doi: 10.1038/s41598-018-33307-z (PMC6175872; doi:10.1038/s41598-018-33307-z)
Supplement: Supplementary file 1 — Supplementary Dataset 1 [file 41598_2018_33307_MOESM1_ESM.docx]

**Supplementary data: Identification of a Novel OX40L+ Dendritic Cell Subset That Selectively Expands Regulatory T cells**

Alejandra Marinelarena*, Palash Bhattacharya*, Prabhakaran Kumar*, Ajay V. Maker*†,

Bellur S. Prabhakar*

**Supplementary Fig-S1**


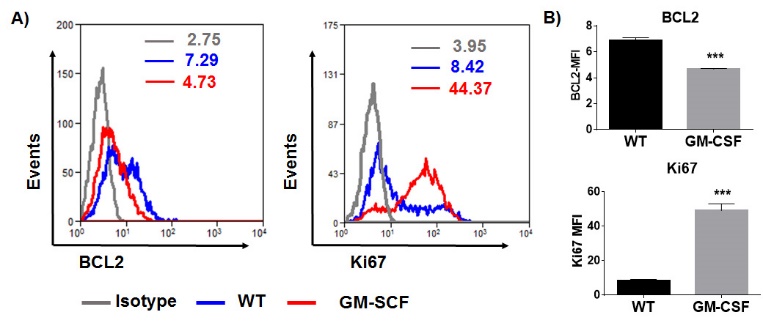


**Supplementary Figure-S1: GM-CSF treatment increased Treg proliferation, rather than survival.** Splenic Tregs from WT and GM-CSF treated mice were analyzed for BCL2 and Ki67 expression. A)Representative overlay histogram show Median Fluorescent Intensity (MFI) values of BCL2 and Ki67 expression in WT (Blue) vs GM-CSF (Red) treated Tregs. B) Bar graph summary of MFI values of BCL2 and Ki67 expression shown in Fig-S1A. Values are expressed as means ± SEM (n=3; ***p < 0.001).
